# Effects of the Czech Propolis on Sperm Mitochondrial Function

**DOI:** 10.1155/2014/248768

**Published:** 2014-07-01

**Authors:** Miroslava Cedikova, Michaela Miklikova, Lenka Stachova, Martina Grundmanova, Zdenek Tuma, Vaclav Vetvicka, Nicolas Zech, Milena Kralickova, Jitka Kuncova

**Affiliations:** ^1^Department of Histology and Embryology, Faculty of Medicine in Pilsen, Charles University in Prague, 301 00 Pilsen, Czech Republic; ^2^Biomedical Centre, Faculty of Medicine in Pilsen, Charles University in Prague, 301 00 Pilsen, Czech Republic; ^3^Institute of Pharmacology and Toxicology, Faculty of Medicine in Pilsen, Charles University in Prague, 301 00 Pilsen, Czech Republic; ^4^Department of Physiology, Faculty of Medicine in Pilsen, Charles University in Prague, 301 00 Pilsen, Czech Republic; ^5^1st Internal Department, Faculty of Medicine and Teaching Hospital in Pilsen, Charles University in Prague, 301 00 Pilsen, Czech Republic; ^6^Department of Pathology, University of Louisville, Louisville, KY 40292, USA; ^7^IVF Centers Prof. Zech - Pilsen, 301 00 Pilsen, Czech Republic

## Abstract

Propolis is a natural product that honeybees collect from various plants. It is known for its beneficial pharmacological effects. The aim of our study was to evaluate the impact of propolis on human sperm motility, mitochondrial respiratory activity, and membrane potential. Semen samples from 10 normozoospermic donors were processed according to the World Health Organization criteria. Propolis effects on the sperm motility and mitochondrial activity parameters were tested in the fresh ejaculate and purified spermatozoa. Propolis preserved progressive motility of spermatozoa in the native semen samples. Oxygen consumption determined in purified permeabilized spermatozoa by high-resolution respirometry in the presence of adenosine diphosphate and substrates of complex I and complex II (state OXPHOS_I+II_) was significantly increased in the propolis-treated samples. Propolis also increased uncoupled respiration in the presence of rotenone (state ETS_II_) and complex IV activity, but it did not influence state LEAK induced by oligomycin. Mitochondrial membrane potential was not affected by propolis. This study demonstrates that propolis maintains sperm motility in the native ejaculates and increases activities of mitochondrial respiratory complexes II and IV without affecting mitochondrial membrane potential. The data suggest that propolis improves the total mitochondrial respiratory efficiency in the human spermatozoa in vitro thereby having potential to improve sperm motility.

## 1. Introduction

Propolis (bee glue) is a natural product that honeybees (*Apis mellifera*) collect from various plants. It is used as building material (filling of cracks and gaps) or for protection against intruders (embalms killed invader insects) [1].

Propolis has been used as a remedy for thousands of years. The term is derived from Greek word* pro*- (meaning in front of) and* polis* (city, community). Its chemical composition depends on the place, time of collection, and plant sources making it highly variable. To date, more than 300 compounds have been detected in various propolis extracts [2, 3]. It is composed mainly of resin (50%) and wax (30%). Other components are pollen (5%), essential and aromatic oils (10%), and minor compounds as flavonoids (quercetin, kaempferol, pinocembrin, apigenin, chrysin, etc.), beta-steroids, terpenes, minerals, and vitamins [1, 4–6]. Propolis is known in folk medicine for its pharmacological effects: antibacterial, antiviral, antifungal, antiparasitic, anti-inflammatory, chemopreventive, immunomodulatory, hepatoprotective, antioxidant, and antitumor [1, 7]. As a result of these effects, over the past 30 years, propolis has been the subject of intense medical studies.

Infertility affects 10–15% of couples of reproductive age and plenty of unanswered questions remain concerning the physiological mechanisms underlying the successful fertility [8]. Male factor contributes to about 50% of cases of infertility and most of them are still idiopathic [9]. More than 90% of male infertility cases are due to low sperm count (oligozoospermia), poor sperm motility (asthenozoospermia), abnormal sperm morphology (teratozoospermia), or all three. The remaining cases of male infertility can be caused by a number of factors including anatomical problems, hormonal imbalances, and genetic defects.

Appropriate sperm motility is fundamental for reproductive success in mammals since the spermatozoa have to travel a relatively long distance through the female reproductive system by active flagellar motion. The energy for the sperm movement in the form of adenosine triphosphate (ATP) is supplied by two metabolic processes: glycolysis taking place in the cytoplasm or oxidative phosphorylation in the mitochondria found only in the sperm midpiece, where 72–80 helically arranged organelles can be detected. However, the relative contributions of both pathways to the sperm motility in different species are a matter of long-standing debate [10, 11]. Nevertheless, recent experimental data suggest that reduced efficiency of the mitochondrial respiratory activity may contribute to the reduced sperm motility. In asthenozoospermic patients, morphological and functional changes in sperm mitochondria have been described [12, 13].

Until now, the effects of propolis on the mitochondrial morphology and function have been studied particularly in the somatic cells. However, the results of these studies are far from uniform showing both stimulation and inhibition of various mitochondrial functions including oxygen consumption, apoptosis, and mitochondrial membrane potential [14–17]. The aim of our study was to assess the effect of ethanolic extract of propolis (EEP) on the human sperm motility, mitochondrial respiratory activity, and membrane potential to test the putative therapeutic potential of this natural product in the treatment of asthenozoospermia.

## 2. Methods

### 2.1. Ethanolic Extract of Propolis Preparation

Propolis was collected using plastic nets in region of West Bohemia (Horni Slavkov—50° 8′17.268^″^ N, 12° 48′48.992^″^ E) in September 2012. Propolis was frozen at −20°C and ground in a mill. The resulting powder (10 g) was mixed at room temperature with 70% ethanol (100 mL) for 24 h and then filtered. The filtrate was then made up to 100 mL with 70% ethanol [18]. The sample was kept in darkness at 4°C until analysis. For experiments with live cells, propolis was further diluted resulting in a final ethanol concentration below 1% which is not toxic to cells [19]. The final concentration of propolis chosen for further experiments was 0.01 mg/mL of the corresponding medium.

### 2.2. High Performance Liquid Chromatography Analysis (HPLC)

Qualitative and quantitative chromatographic analyses of phenolics were performed on a HPLC system equipped with a binary pump (Waters 1525), Waters 717 plus Autosampler, and dual UV/VIS detector 2487. Separation was performed on a Symmetry C18 column, particle size 5 *μ*m (150 mm × 4.6 mm), using a mobile phase of 0.08% acetic acid in methanol (A) and 0.1% acetic acid and 10% methanol in water (B). The gradient was 10–47% A (25 min), 47% A (25–40 min), 47–70% A (40–70 min), and 70–100% A (70–80 min) at a flow rate of mobile phase 0.5 mL/min. Injection volume was 10 *μ*L, and column temperature was 30°C.

Spectrophotometric detection was conducted at 280 nm and 330 nm. Identification of polyphenolic compounds was achieved by comparison of retention times with those of commercial pure compounds. All standards were dissolved in dimethyl sulfoxide (Sigma-Aldrich; St. Louis, USA) to give 10 mmol/L standard solutions. Calibration standards were prepared by dilution of the standard solution in ethanol.

Quantitative analysis was carried out by external standard method. Calibration curves showed a linear response of *R* ^2^ > 0.97 over a concentration range of 5–100 *μ*mol/L. Before the HPLC analysis, the EEP was filtered on teflon syringe microfilter Separon 0.45 *μ*m. The propolis extract was diluted one hundred times for HPLC analysis.

Phenolic compounds were purchased from Sigma-Aldrich (St. Louis, USA) (apigenin, chrysin, genistein, kaempferol, luteolin, naringenin, pinocembrin, galangin and phenolic acids: caffeic, p-coumaric, t-ferulic, t-cinnamic, benzoic, and gallic acid and caffeic acid phenethyl ester). Vanillin was purchased from Merck (Darmstadt, Germany).

### 2.3. Sperm Sample Preparation

The study design was approved by the Local Ethics Committee of the University Hospital in Pilsen and a written informed consent was obtained from each of the 10 participants included in the study (mean age 24.2 years, SEM ± 2.8).

Ejaculates were collected after 3 days of sexual abstinence in IVF Center Prof. Zech, Pilsen. Semen samples were evaluated by an experienced employee. After liquefaction, they were analyzed according to the World Health Organization criteria 2010 [20]. We investigated semen volume and, under the microscope with phase-contrast optics at magnification ×200, concentration of spermatozoa, motility of spermatozoa and pathologies. Sperm motility was assessed at room temperature in Makler counting chamber. Two hundred spermatozoa per replicate were classified into three motility categories (progressive, nonprogressive, and immotile sperm cells). All samples were considered normozoospermic ejaculates (Table 1).

Fresh ejaculate (0.1 mL) was subjected to experiment with propolis. Propolis or ethanol only (1 *μ*L) was added to 0.1 mL of fresh ejaculate (final concentration of propolis was 0.01 mg/mL) and sperm motility was evaluated after 60 minutes.

The remaining sample was prepared by gradient separation technique and used for experiment with polarographic oxygraph (Oroboros, Innsbruck, Austria) and sperm flow cytometry evaluation. Sperm number after separation was also determined in the Makler counting chamber.

### 2.4. The Sperm Density Gradient Separation Technique

The sperm was separated and purified. This was performed using gradient solution media SpermGrad medium (SGm, Vitrolife, Sweden) and SpermRinse medium (SRm, Vitrolife, Sweden). SpermGrad medium was diluted to 90% (0.15 mL SGm : 1.35 mL SRm), 70% (0.09 mL SGm : 0.21 mL SRm) and 50% (0.15 mL SG : 0.15 mL SRm). Into a conical tube 1.5 mL 90%, 0.3 mL 70% and 0.3 mL of a 50% gradient media were layered. Full ejaculate was added and the sample was centrifuged for 20 minutes at 300 g. After removal of the supernatant, 8 mL SRm was added and then the sample was centrifuged 8 minutes at 300 g. After removal of supernatant, the sample was evaluated for the concentration and motility of spermatozoa and ready for injection into the oxygraph.

### 2.5. High-Resolution Respirometry

Oxygen consumption by purified spermatozoa was measured at 36°C in 2 mL glass chambers of oxygraph Oroboros (Oroboros, Innsbruck, Austria) connected to the computer with DatLab software for data acquisition and analysis (Oroboros, Innsbruck, Austria). The oxygen flux was calculated as a negative time derivative of the oxygen concentration. All values of oxygen fluxes were corrected for instrumental and chemical background measured in separate experiments performed in the same medium without human gametes.

**Figure 1:**
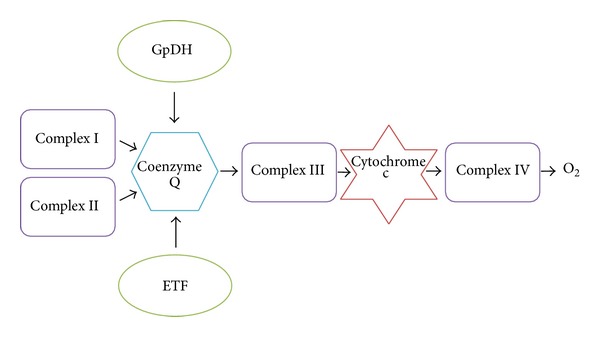
A scheme of an electrotransport convergent system. System is situated on the inner mitochondrial membrane. GpDH = glucose-6-phosphate dehydrogenase; complex I = NADH-Q reductase; complex II = succinate-Q oxidoreductase; complex III = cytochrome reductase; complex IV = cytochrome oxidase; ETF = electron-transporting flavoprotein; O_2_ = oxygen.

The medium consisting of 0.5 mmol/L ethylene glycol tetraacetic acid, 3 mmol/L MgCl_2_ *·*6H_2_O, 60 mmol/L K-lactobionate, 20 mmol/L taurine, 10 mmol/L KH_2_PO_4_, 20 mmol/L HEPES, 110 mmol/L sucrose, and 1 g/L albumin essentially fatty acid free [21] was stirred at 750 rpm and equilibrated for 60 min with air. After equilibration, oxygen concentration in the chamber corresponded to its concentration in the atmospheric air and solubility in the medium (0.92). The chambers were then closed and the samples of intact spermatozoa were injected into the chambers using Hamilton syringe. Into one of two chambers recording in parallel, propolis (0.01 mg/mL) was injected and the samples were further incubated at 36°C for 20 min. The spermatozoa cell membrane was permeabilized with digitonin (Sigma-Aldrich, St. Louis, USA; 5 *μ*g/mL) and combination of substrates, inhibitors, and uncouplers was sequentially injected into the chambers to measure the respiration through different segments of the electron transport system (Figure 1). (1) Resting respiration with substrates providing electrons to complex I malate (2 mmol/L) and glutamate (10 mmol/L) was measured as a state S2 (nonphosphorylating LEAK state, L_N_). (2) Active respiration was induced by 5 mmol/L adenosine diphosphate (ADP; state S3 or OXPHOS). (3) Oxygen consumption was further measured with pyruvate (5 mmol/L) and a substrate of electron transfer flavoprotein (ETF) palmitoyl carnitine (20 *μ*mol/L). (4) Integrity of the mitochondrial inner membrane was checked with cytochrome c (10 *μ*mol/L). (5) Mitochondrial respiration was then increased by succinate, complex II substrate (10 mmol/L). (6) State LEAK was induced again by inhibition of ATP-synthase oligomycin (2 *μ*g/mL). (7) Maximum capacity of the electron-transporting system (state S3u or ETS) was reached by titration of uncoupler trifluorocarbonylcyanide phenylhydrazone (FCCP; 0.05 *μ*mol/L titration steps). (8) After addition of a complex I inhibitor rotenone, the oxygen flux corresponded to maximum capacity of the electron-transporting system with the complex II only. (9) Then, antimycin A (2.5 *μ*mol/L), a complex III inhibitor was injected into the chambers to measure residual oxygen consumption (ROX). (10) N,N,N′,N′-tetramethyl-p-phenylenediamine dihydrochloride (TMPD; 0.5 mmol/L) and ascorbate (2 mmol/L) were injected simultaneously for respirometric assay for cytochrome c oxidase (C IV) activity. In the results, oxygen fluxes recorded in the individual titration steps were corrected for residual oxygen consumption.

The dose-response relationship between the propolis concentration and sperm respiratory activity was tested in another set of experiments, where the final concentrations of propolis 0.001, 0.005, and 0.01 mg/mL were used. Higher dose of propolis was not tested as in experiments running in parallel, higher concentrations of propolis in the incubation medium (0.03 and 0.05 mg/mL) were toxic for mouse embryonic stem cells reducing their growth, survival, and proliferation (unpublished observation).

### 2.6. Permeability of Cell Membrane in Sperm

Sperm was treated with the impermeable fluorescent dye propidium iodide to check cell membrane permeability for substrates, inhibitors, and uncouplers after treatment by digitonin. Final concentration of propidium iodide was 1 *μ*g/mL.

### 2.7. Sperm Flow Cytometry Evaluation

Mitochondrial membrane potential (ΔΨ*m*) was determined with MitoProbe JC-1 Assay Kit (Life Technologies). Each sperm sample was resuspended in 37°C warm phosphate-buffered saline (PBS) at approximately 1 × 10^6^ cells/mL. Propolis (1 *μ*L) was added to test samples (100 *μ*L) to reach final concentration 0.01 mg/mL. Controls remained without intervention. Samples were incubated in propolis for 60 min and after that were washed with PBS. JC-1 (10 *μ*L of 200 *μ*mol/L) was added for 20 min incubation (37°C, 5% CO_2_). Wash with PBS followed (5 min, 1500 rpm). Samples were resuspended in 500 *μ*L PBS and measured on BD FACS CANTO II cytometer (BD Biosciences, New Jersey, USA). Analysis was performed with BD FACS Diva software with 488 nm excitation using emission filters appropriate for Alexa Fluor 488 dye and R-phycoerythrin.

### 2.8. Citrate Synthase Activity

Mitochondrial content in the samples aspirated from each oxygraph chamber was assayed by determination of the citrate synthase activity [22, 23]. The assay medium consisted of 0.1 mmol/L 5,5-dithio-bis-(2-nitrobenzoic) acid, 0.25% Triton-X, 0.5 mmol/L oxalacetate, 0.31 mmol/L acetyl coenzyme A, 5 *μ*mo/L EDTA, 5 mmol/L triethanolamine hydrochloride, and 0.1 mol/L Tris-HCl, pH 8.1 [22]. Two hundred microliters of the mixed and homogenized chamber content was added to 800 *μ*L of the medium. The enzyme activity was measured spectrophotometrically at 412 nm and 30°C over 200 s and expressed in mIU per 10^7^ cells.

### 2.9. Data Analysis and Statistics

Results are presented as mean ± SEM. Statistical differences were analyzed using software package STATISTICA Cz, 8 (StatSoft Inc., Prague, Czech Republic). After testing for the normality of distribution and homogeneity of variances, comparisons were made using Student's *t*-test, Wilcoxon signed-rank test and analysis of variance (ANOVA) with post hoc tests corrected for multiple comparisons by Bonferroni's method. The results were considered significantly different when *P* < 0.05.

## 3. Results

### 3.1. HPLC Analysis

**Figure 2:**
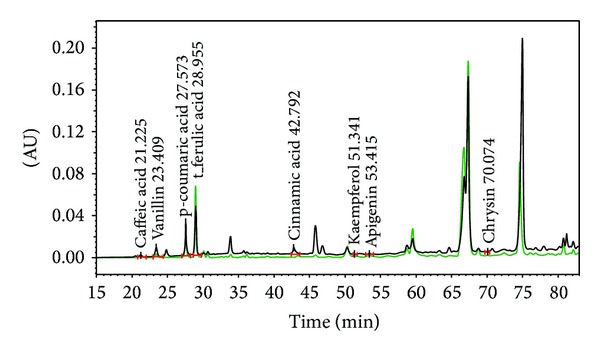
HPLC chromatogram of the Czech propolis extract and the identified compounds/retention time (*λ* = 280 nm—black line and *λ* = 330 nm—green line).

Analyzing the propolis by the HPLC, we were able to identify compounds as t-ferulic acid, p-coumaric acid, vanillin, caffeic acid, t-cinnamic acid, kaempferol, apigenin, and chrysin. Although we analyzed standards of gallic acid, benzoic acid, quercetin, naringenin, luteolin, genistein, pinocembrin, galangin, and caffeic acid phenethyl ester, they were not identified in our propolis sample. Chromatogram of ethanolic extract of the Czech propolis is presented in Figure 2. Detailed results with concentrations of observed substances are shown in Table 2.

### 3.2. Semen Parameters and Effect of Propolis on Sperm Motility in Fresh Ejaculate

**Figure 3:**
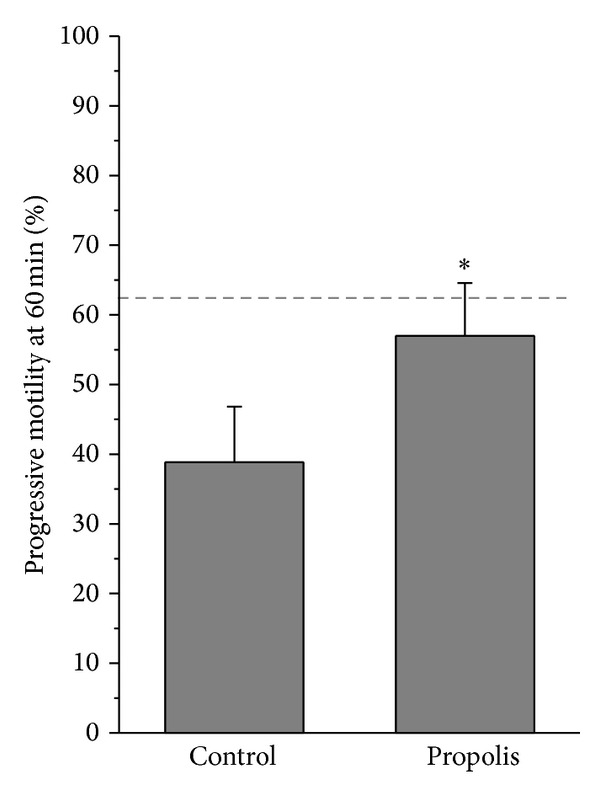
Effect of propolis on sperm motility. Columns represent progressive motility of spermatozoa in native ejaculates after 60 min incubation with propolis extract (mean ± SEM). Dashed line = progressive motility at time 0. *P < 0.05, compared to the respective control value.

Ten healthy men were included in this study. The mean age was 24.2 years. The general sperm characteristic of the normozoospermic men after ejaculation is shown in Table 1. The effect of propolis on human sperm motility after incubation for 60 minutes is presented in Figure 3. Propolis preserved the progressive motility of spermatozoa in the native semen samples, since the percentage of the progressively motile spermatozoa after incubation with propolis remained nearly the same as in the fresh samples, whereas in the ejaculates without propolis, the progressive motility significantly declined with time (*P* = 0.028). Ethanol alone had no negative effect on sperm motility.

### 3.3. High Resolution Respirometry

**Figure 4:**
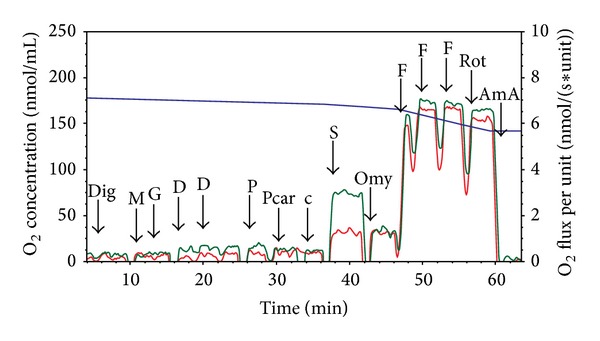
Substrate-uncoupler-inhibitor titration protocol in human spermatozoa. Substrate-uncoupler-inhibitor titration (SUIT) protocol with substrates for complex I, complex II, and electron-transferring flavoprotein (ETF) in human spermatozoa. Red line = oxygen flux expressed per IU citrate synthase activity in the control sample, green line = oxygen flux expressed per IU citrate synthase activity in the sample treated with propolis 0.01 mg/mL, and blue line = oxygen concentration in the oxygraph chamber. Dig = digitonin, M = malate, G = glutamate, D = ADP, P = pyruvate, Pcar = palmitoylcarnitine, c = cytochrome c, S = succinate, Omy = oligomycin, F = FCCP, Rot = rotenone, and AmA = antimycin A.

**Figure 5:**
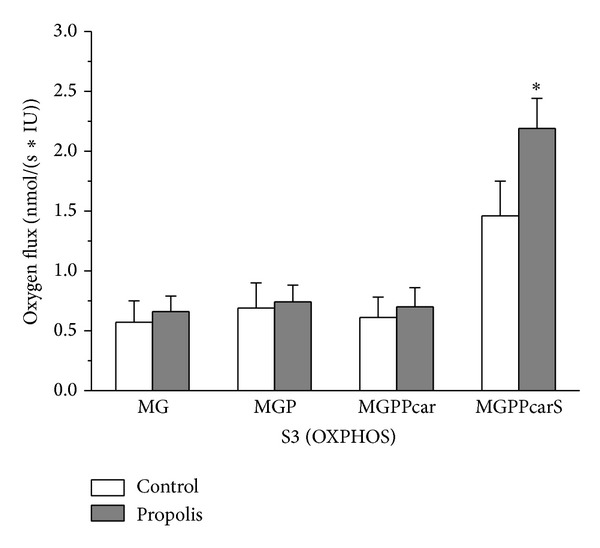
Oxygen consumption rates in the state S3 (OXPHOS) measured under control conditions and with propolis. Oxygen consumption rates in the state S3 (OXPHOS) measured under control conditions (control) and with propolis extract added (propolis) in the presence of ADP and substrates providing electrons to complex I, ETF, and complex II. M = malate, G = glutamate, P = pyruvate, Pcar = palmitoylcarnitine, and S = succinate. Oxygen fluxes were corrected for residual oxygen consumption and expressed per IU citrate synthase activity. *P < 0.05, compared to the respective control value.

**Figure 6:**
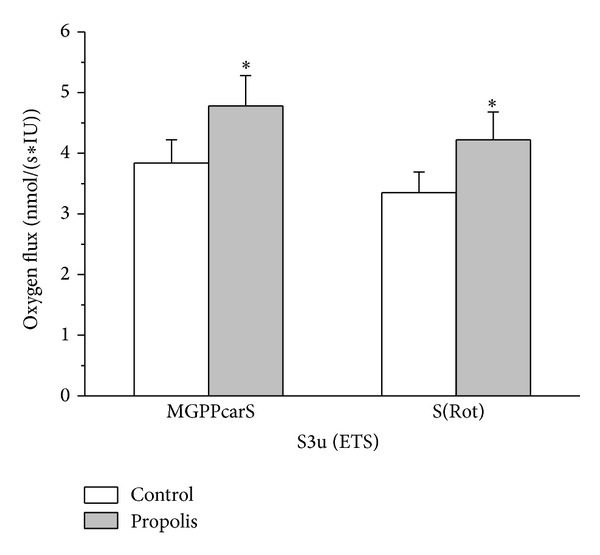
Oxygen consumption rates in the state S3u in permeabilized spermatozoa. Oxygen consumption rates in the state S3u (ETS) in permeabilized spermatozoa measured under control conditions (control) and with propolis extract added (propolis) in the presence of FCCP and substrates providing electrons to complex I, ETF, and complex II. M = malate, G = glutamate, P = pyruvate, Pcar = palmitoylcarnitine, S = succinate, and complex I inhibitor rotenone (Rot). Oxygen fluxes were corrected for residual oxygen consumption and expressed per IU citrate synthase activity. *P < 0.05, compared to the respective control value.

Representative traces of the oxygen consumption in permeabilized spermatozoa with and without propolis 0.01 mg/mL are depicted in Figure 4. Oxygen consumption of intact spermatozoa (0.13 ± 0.01 nmol/(s*·*IU)) was significantly enhanced by propolis (0.27 ± 0.03 nmol/(s*·*IU); *P* = 0.006). After permeabilization with digitonin, state 2 determined in the presence of malate and glutamate was 0.19 ± 0.04 nmol/(s*·*IU) in the control samples and it was significantly higher in the propolis-treated sperm samples (0.29 ± 0.05 nmol/(s*·*IU); *P* = 0.014). State 3, that is, oxygen consumption during oxidative phosphorylation, determined in the presence of ADP and substrates of the complex I, ETF, and complex II is depicted in Figure 5. Propolis significantly increased (by ~50%) S3_I+II_ oxygen flux (*P* = 0.003) suggesting increased activity of the complex II. Propolis did not influence state 4 induced by oligomycin that reached 0.81 ± 0.07 and 0.94 ± 0.09 nmol/(s*·*IU) in the control and propolis-treated spermatozoa, respectively. Maximum capacity of the electron-transporting system (state S3u) tested for both complexes I and II and in the presence of the complex I inhibitor rotenone was significantly higher after propolis administration (Figure 6). In measurements with TMPD + ascorbate, complex IV respiration was 12.16 ± 2.58 nmol O_2_/(s*·*IU) in the control samples and it was significantly enhanced by propolis to 15.4 ± 3.19 nmol O_2_/(s*·*IU). Absolute ethanol alone (medium for propolis, oligomycin, antimycin A, FCCP, and rotenone) in the volume up to 10 *μ*L did not influence respirometric parameters measured with substrates of complexes I, II, and ETF in the presence of ADP.

Flux control ratios were calculated to estimate the relative efficiency of individual interventions and coupling state of the sperm mitochondria. The ratio OXPHOS_I+II_/OXPHOS_I_ was significantly higher in the propolis-treated samples (3.53 ± 0.42) compared to controls (2.8 ± 0.28) suggesting increased efficiency of coupled respiration when complex II was stimulated by succinate. The LEAK control ratio is the ratio of LEAK respiration and ETS capacity; it reached 0.25 ± 0.02 in the control samples and it significantly decreased after propolis to 0.21 ± 0.02 (*P* = 0.024).

**Figure 7:**
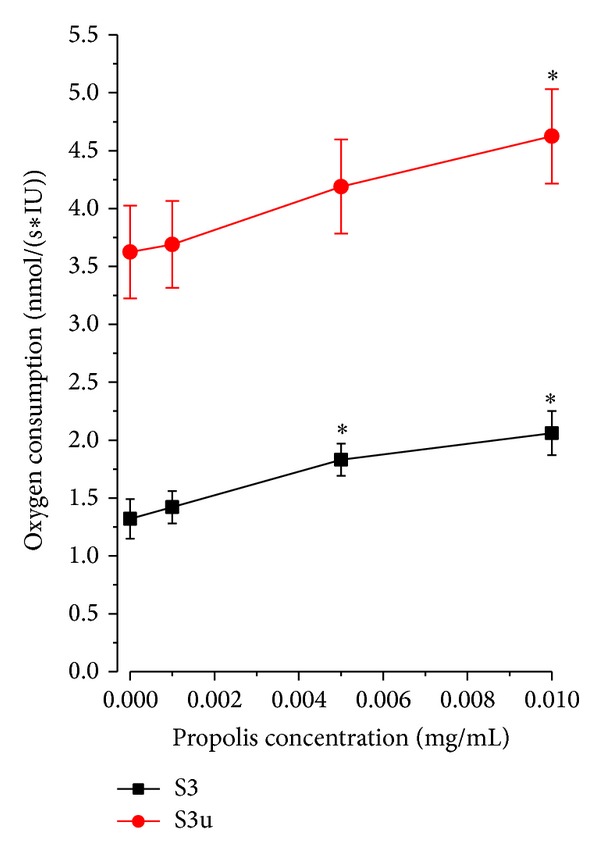
Dose response relationship between oxygen consumption and final propolis concentration. State S3 (OXPHOS) = oxygen consumption rate in permeabilized spermatozoa measured with malate, glutamate, pyruvate, succinate, and ADP. State S3u = oxygen consumption rate in permeabilized spermatozoa measured after sequential addition of malate, glutamate, ADP, pyruvate, succinate, and FCCP. Oxygen fluxes were corrected for residual oxygen consumption and expressed per IU citrate synthase activity. *P < 0.05, compared to the respective value without propolis.

The dose-response relationship tested in separate experiments is shown in Figure 7 for state S3 (OXPHOS), where glutamate, malate, pyruvate, succinate, and ADP were present in the medium, and for state S3u (ETS), where mitochondria where uncoupled by FCCP. Propolis dose-dependently increased oxygen consumption, although the extent of its effect was greater for state S3 than for state S3u.

### 3.4. Permeability of Cell Membrane in Sperm

**Figure 8:**
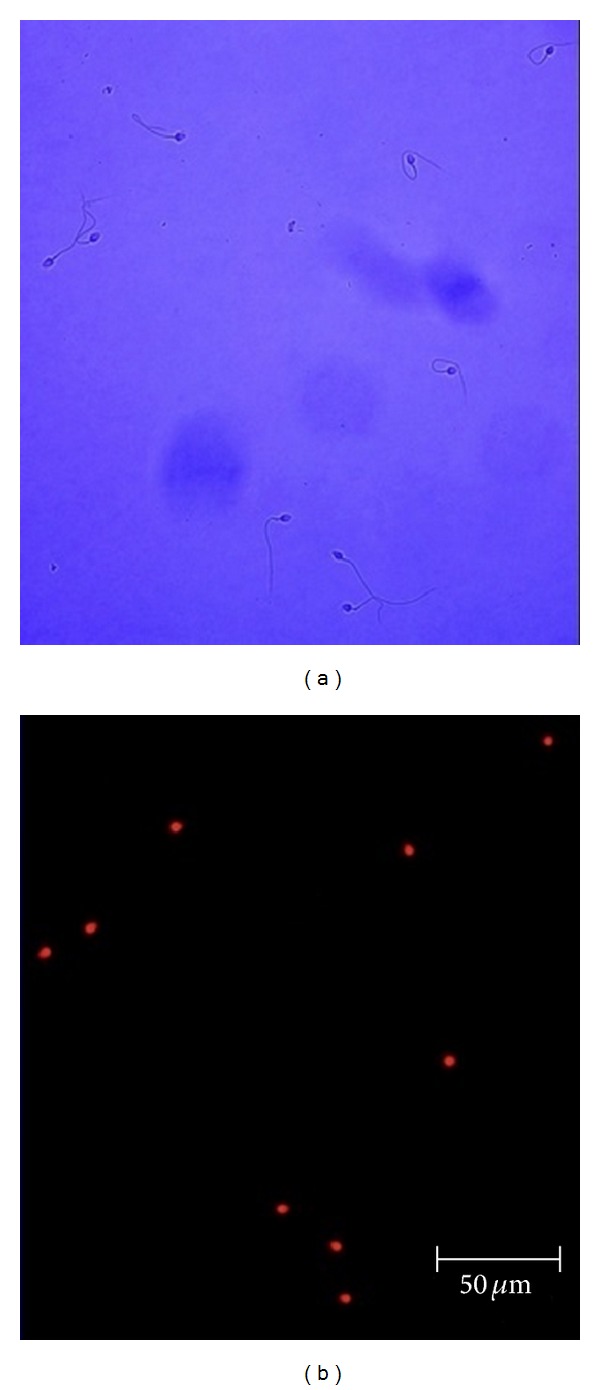
Effect of digitonin treatment on spermatozoa. (a) Micrograph of human spermatozoa and (b) fluorescent micrograph of the same optical field after treatment with the nonpermeable nuclear dye propidium iodide.

Addition of 5 *μ*g/mL of digitonin made the sperm cell membrane permeable to dye propidium iodide and for the substrates, inhibitors, and uncouplers required to determine high resolution respirometry (Figure 8).

### 3.5. Sperm Flow Cytometry Evaluation

**Figure 9:**
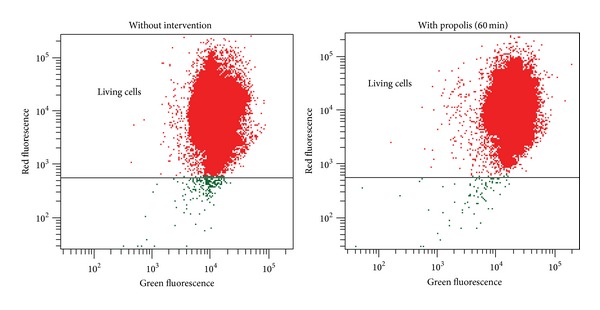
Typical double fluorescence dot plots of flow cytometry analysis. Mitochondrial membrane potential was determined with MitoProbe JC-1 Assay Kit (Life Technologies). R-phycoerythrin = red fluorescence, normal cells; Alexa Fluor 488 dye = green fluorescence, reduced mitochondrial membrane potential.

Depolarization of the mitochondrial membrane is a sensitive indicator of mitochondrial damage. JC-1 is a membrane-permeable fluorescent probe aggregating in the mitochondrial matrix and then emitting red fluorescence, if ΔΨ*m* is high. In case of mitochondrial depolarization, the monomeric form of JC-1 cannot accumulate in the mitochondrial matrix and produce green fluorescence in the cytoplasm. In the spermatozoa, loss of ΔΨ*m* could serve as a marker of early apoptosis and sperm dysfunction [24]. In our experiments, incubation of the sperm samples with propolis (60 minutes) had no significant effect on ΔΨ*m* (Figure 9). In the control samples, the percentage of cells with high ΔΨ*m* was 98.43 ± 1.07% and it even slightly increased in the propolis-treated spermatozoa reaching 99.33 ± 0.25%.

### 3.6. Citrate Synthase Activity

In the control samples, citrate synthase activity was 6.58 ± 0.84 mIU/10^7^ cells and it was not significantly affected by propolis (6.08 ± 0.84 mIU/10^7^ cells) and by the substances (substrates, inhibitors, media) added during the respirometric measurements, as tested in separate controls.

## 4. Discussion

This is the first study showing chemical composition of propolis collected in the Czech Republic. Bee glue has a very complex chemical composition that depends on a number of factors including diversity of plants and geographical location from which bees collect it. Although the region of collection of the tested sample of propolis is situated in the northern temperate zone where the “poplar” type propolis is a typical bee product [3], the tree structure within the mentioned locality is composed of coniferous trees (90%), birch (6%), alder (2%), beech (1%), and oak (1%) [25]. However, the tested sample contained chrysin, the reference flavonoid in poplar propolis, and phenolic acids and other flavonoids typically found in bee glue extracts originating from similar geographical regions and reported to be responsible for various beneficial effects of propolis [26]. Among them, ferulic acid, coumaric acid, and kaempferol were present in the highest concentrations in the Czech propolis extract. Caffeic acid phenethyl ester, naringenin, and quercetin—substances frequently contained in the poplar propolis [2, 3] and widely tested for their anti-inflammatory, anticancer, and antioxidant activities in recent years [27]—have not been detected in the Czech propolis. The factors that could contribute to the above mentioned differences could include not only diversity of plant species growing around the hive, but also season, illumination, altitude, collector type, and food availability [28]. It should be noted that the search for a single substance or a particular substance class that could be responsible for the beneficial actions of propolis has not been successful to date. Scientific studies demonstrated that biological activity of propolis could be almost identical (i.e., antimicrobial, antitumor, antioxidant, anti-inflammatory, etc.) in samples from different climatic zones and of completely different chemical composition [3]. Most probably, a combination of substances is essential for the biological activity of bee glue [29].

The present study describes the effects of propolis on the sperm motility, mitochondrial membrane potential, and mitochondrial respiratory activity assessed by high-resolution respirometry allowing determination of individual respiratory states in a sequential manner on the same sample, thus allowing complex evaluation of the mitochondrial function close to the situation in vivo. In addition, high sensitivity of the method enables determination of respirometric activity of individual mitochondrial complexes in a relatively small amount of cells [30]. In our experiments, we have used the purified spermatozoa that were subjected to membrane permeabilization by mild nonionic detergent digitonin, the dose of which was carefully titrated to permeabilize the cell membrane without damaging the mitochondrial function. To date, oxygen consumption by human sperm mitochondria has been determined mostly by traditional oxygraphy in the germ cells subjected to hypotonic swelling [13, 31–33]. However, in the spermatozoa of several mammalian species, hypotonic challenge could substantially influence the activity of various protein kinases (PK), including PKA, PKC, and protein-tyrosine kinase via osmosensitive K^+^ and Cl^−^ channels in the process called regulatory volume decrease requiring energy supply [34].

The major finding of the present study is that propolis enhances the activity of mitochondrial respiratory complexes II and IV without affecting the coupling of the electron transport to ATP synthesis and/or mitochondrial membrane potential in permeabilized human spermatozoa in vitro. In the coupled state, propolis enhanced oxygen consumption with complex I and complex II substrates by ~50%. This increase was attributed to complex II, since the activity of complex I alone was not affected by propolis. In addition, the ratio *P*_I+II_/*P*_I_ was significantly higher in the propolis-treated samples. Similarly, in the uncoupled state (S3u, E), oxygen consumption was significantly (by ~25%) higher after propolis, both in the presence of substrates of complexes I and II and after inhibition of complex I by rotenone. Activity of the complex IV was increased to the same extent (~27%).

The data available on the effects of propolis and its major compounds on mitochondrial respiration or activity of individual mitochondrial enzymes involved in the electrontransport convergent system are scarce. The most frequent finding is that this effect is negligible in normal somatic cells (cardiomyocytes, neurons, and hepatocytes), but becomes beneficial in the cells challenged by toxic stimuli where oxidative phosphorylation is compromised [16, 35, 36]. In contrast, in various types of tumor cells, propolis extract or its constituents inhibit oxidative phosphorylation and trigger release of cytochrome c and subsequent apoptosis [15, 17]. In view of this, the action of propolis on the cellular respiration is not easily predictable and depends on the cell type.

Some studies suggested that propolis or its phenolic constituents might influence mitochondrial membrane potential via increased permeability of the inner mitochondrial membrane [14]. This issue was addressed in the experiments with MitoProbe JC-1 Assay Kit that clearly showed that in the human spermatozoa, mitochondrial membrane potential was not affected by propolis. In addition, oligomycin-induced respiratory state (LEAK) that reflects compensation for the proton leak, proton slip, electron slip, and cation cycling [30] was not affected by propolis. Thus, the decrease in the LEAK control ratio after propolis could be attributed to the increased efficiency of the electron transport through complex II. The putative molecular mechanism of complex II activation was suggested in study of Cimen et al. [37], where propolis constituent kaempferol increased deacetylation of succinate dehydrogenase thus increasing its activity.

To date, the effects of propolis or propolis compounds on the sperm characteristics have been rarely studied. The whole propolis extract was used in studies conducted by Yousef and collaborators on rats and rabbits that were treated with propolis for 10 to 12 weeks [38, 39]. Administration of propolis resulted in an increased sperm count and motility, plasma testosterone levels, and a decreased dead and abnormal sperm count. A single study documented positive effect of the propolis compound chrysin on the sperm motility, sperm concentration, and serum testosterone levels [40]. Ferulic acid was also reported to enhance sperm motility and viability [41]. All these findings were attributed to the activity of propolis as antioxidant and none of these studies dealt with the action of propolis or its constituents on mitochondrial energy production. Our study describes a novel beneficial effect of propolis on the sperm characteristics.

Recent experimental evidence suggests that oxidative phosphorylation in the human spermatozoa plays a crucial role in gaining energy for the sperm motility and indicates that asthenozoospermia might be related to the impaired mitochondrial functionality [42]. High-resolution respirometry could provide new data in search for substances that could positively affect human sperm motility and thus improve sperm fertilizing ability. In addition, detailed analysis of respiratory efficiency of individual mitochondrial enzymatic complexes under coupled and uncoupled conditions could provide better insight into pathophysiology of asthenozoospermia.

## 5. Conclusions

This study demonstrates, for the first time, that ethanolic extract of propolis increases activities of mitochondrial respiratory complexes II and IV without affecting mitochondrial membrane potential. The obtained data suggest that propolis improves the total mitochondrial respiratory efficiency in the human spermatozoa in vitro thereby having potential to improve the sperm motility.

## Figures and Tables

**Table 1:** Main sperm parameters of the normozoospermic men (±SEM).

Parameters	Normozoospermic men (*n* = 10)
Volume (mL)	3.18 ± 0.26
Concentration (×10^6^/mL)	81.22 ± 13.97
Progressive motility (%)	62.5 ± 4.4
Pathology morphology (%)	42.00 ± 2.26
Concentration after separation (×10^6^/mL)	158.44 ± 18.09
Progressive motility after separation (%)	86.43 ± 2.10

**Table 2:** Analysis of the ethanolic extract of propolis by the HPLC.

Compound	Rt [min]	Concentration [mg/L of EEP]
Gallic acid	6.0	n.d
Caffeic acid	21.2	**65 ± 11**
Vanillin	23.4	**65 ± 11**
p-Coumaric acid	27.5	**231 ± 10**
t-Ferulic acid	28.9	**514 ± 15**
Benzoic acid	33.6	n.d
Quercetin	42.3	n.d
t-Cinnamic acid	42.7	**29 ± 1**
Naringenin	43.2	n.d.
Luteolin	44.7	n.d
Genistein	45.6	n.d
Kaempferol	51.3	**101 ± 45**
Apigenin	53.4	**73 ± 8**
Chrysin	70.0	**36 ± 5**
Pinocembrin	65.1	n.d.
Galangin	73.1	n.d.
CAPE	71.4	n.d.

(n.d. = nondetected; CAPE = caffeic acid phenethyl ester).
